# Bronchial hyperresponsiveness in an adult population in Helsinki: decreased FEV_1_, the main determinant

**DOI:** 10.1111/j.1752-699X.2012.00279.x

**Published:** 2013-01

**Authors:** Maria Juusela, Paula Pallasaho, Seppo Sarna, Päivi Piirilä, Bo Lundbäck, Anssi Sovijärvi

**Affiliations:** 1 Department of Clinical Physiology and Nuclear Medicine, Laboratory of Clinical Physiology, Helsinki University HospitalsHelsinki, Finland; 2 Control of Hypersensitivity Diseases, Finnish Institute of Occupational HealthHelsinki, Finland; 3 Department of Public Health, Helsinki UniversityHelsinki, Finland; 4 Krefting Research Centre, Sahlgrenska Academy, University of GothenburgGothenburg, Sweden

**Keywords:** bronchial hyperresponsiveness, histamine, respiratory symptoms, sensitization

## Abstract

**Introduction:**

Bronchial hyperresponsiveness (BHR) elevates the risk for development of respiratory symptoms and accelerates the decline in forced expiratory volume in the first second (FEV_1_). We thus aimed to assess the prevalence, determinants and quantity of BHR in Helsinki.

**Objectives:**

This study involved 292 randomly selected subjects age 26–66 years, women comprising 58%.

**Methods:**

Following a structured interview, a spirometry, a bronchodilation test, and a skin-prick test, we assessed a bronchial challenge test with inhaled histamine using a dosimetric tidal breathing method. Results included the provocative dose inducing a decrease in FEV_1_ by 15% (PD_15_FEV_1_) and the dose-response slope. For statistical risk factor-analyses, the severity of BHR was considered; PD_15_ values ≤1.6 mg (BHR) and ≤0.4 mg [moderate or severe BHR (BHR_ms_)] served as cut-off levels.

**Results:**

BHR presented in 21.2% and BHR_ms_ in 6.2% of the subjects. FEV_1_ < 80% of predicted [odds ratio (OR) 4.09], airway obstruction (FEV_1_/forced vital capacity < 88% of predicted) (OR 4.33) and history of respiratory infection at age <5 (OR 2.65) yielded an increased risk for BHR as ORs in multivariate analysis. For BHR_ms_, the determinants were decreased FEV_1_ below 80% of predicted (OR 27.18) and airway obstruction (OR 6.16). Respiratory symptoms and asthma medication showed a significant association with BHR.

**Conclusions:**

Of the adult population of Helsinki, 21% showed BHR to inhaled histamine. The main determinants were decreased FEV_1_ and airway obstruction. Quantitative assessment of BHR by different cut-off levels provides a tool for characterization of phenotypes of airway disorders in epidemiologic and clinical studies.

Please cite this paper as: Juusela M, Pallasaho P, Sarna S, Piirilä P, Lundbäck B and Sovijärvi A. Bronchial hyperresponsiveness in an adult population in Helsinki: decreased FEV_1_, the main determinant. *Clin Respir J* 2013; 7: 34–44.

## Introduction

Bronchial hyperresponsiveness (BHR), a measure of functional airway disturbance in asthma, is a common consideration in epidemiologic studies (1–5). In Finland, the prevalence of physician-diagnosed asthma in adults has increased to 7% (6, 7), but epidemiologic data on BHR and its associations are available only from a selected study cohort from near the Arctic Circle (8).

A typical sign of asthma is inducible airway obstruction. BHR is associated with inflammation of the airways (9), geometric changes in the airway-tree (10, 11), heterogeneity of ventilation (12, 13) and ventilation-perfusion mismatch in the lungs (14), which links these research findings to asthma and bronchial provocation tests. Smoking (15, 16), obesity (17) and aging (18) are involved in an abnormal decline in ventilatory function and in BHR. Because recent epidemiologic studies have concluded that increased airway responsiveness is associated with an enhanced risk for respiratory symptoms and accelerated decline in forced expiratory volume in the first second (FEV_1_) (19, 20), we aimed to assess the magnitude of BHR in an adult population and the determinants of BHR of different magnitudes. BHR has not previously been assessed by precise methods (21) in a general population, with risk factor analysis for different provocative dose inducing a decrease in FEV_1_ by 15% (PD_15_FEV_1_) cut-off levels. Seldom are factors such as health history, allergic sensitization and lung-function measurements included in multivariate analysis for assessment of the determinants of BHR, as here (19, 20, 22).

Our aim was to determine the prevalence of BHR in a general adult population in Helsinki and to assess the determinants of increased BHR in general (BHR) and of moderate or severe BHR (BHR_ms_) in relation to asthma, airway obstruction, ventilatory function, respiratory symptoms, smoking and allergic sensitization, with data from structured questionnaires, flow-volume spirometry studies and skin-prick tests (SPTs). The present study is a part of the epidemiologic (FinEsS) study in which in a longitudinal setting, follow-up studies have been in progress in Finland, Estonia and Sweden since 1996.

## Materials and methods

### Study cohort and subjects

**Figure 1:**
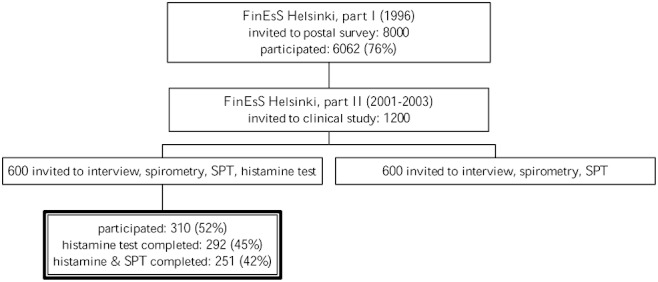
Flow chart for the study cohort. SPT, skin-prick test.

The study involved 292 randomly selected subjects who had taken part in an initial postal questionnaire survey in Helsinki in 1996 (6). Fig. 1 illustrates the flow chart of the study cohort.

Of those who responded, a randomly selected sample of 1200 subjects was invited to the clinical studies in 2001–2003 (23). Of those 1200, for 600 subjects, the protocol included a histamine challenge test. The final participation rate for the present study was 45% (*n* = 292). The Helsinki University Central Hospital Ethics Committee approved the study, with all patients giving written informed consent.

The age range was 26–66 years (women 58%), mean 47. The baseline FEV_1_ of those studied ranged from 62% to 129% of predicted (Finnish population values) (24). For 32 subjects (11%), FEV_1_ was below the lower normal limit (of the predicted <80%), and for 37 (13%), FEV_1_/forced vital capacity (FVC) was below normal (<88% of predicted). See demographic data in Tables 1 and 2.

**Table 1:** Demographic data of the study population with completed histamine tests; values are given as mean ± standard deviation and range (*n* = 292). Forced expiratory volume in the first second (FEV_1_) and forced vital capacity (FVC) values were obtained from 291 subjects. Predicted values according to Viljanen *et al*. (24)

	Men (*n* = 123)	Women (*n* = 169)	Total (*n* = 292)
Age (years)	45.2 ± 9.5 (28–65)	47.3 ± 10.6 (26–66)	46.4 ± 10.2 (26–66)
Height (m)	1.74 ± 0.06 (1.61–1.86)	1.63 ± 0.07 (1.46–1.74)	1.69 ± 0.08 (1.46–1.86)
Weight (kg)	80.0 ± 12.6 (43–110)	70.6 ± 13.8 (48–105)	75.6 ± 14.0 (43–110)
Body mass index	26.50 ± 4.37 (17.90–44.87)	25.98 ± 6.04 (17.10–55.13)	26.20 ± 5.40 (17.10–55.13)
FEV_1_ (L)	4.06 ± 0.70 (2.35–5.90)	2.87 ± 0.51 (1.71–4.50)	3.37 ± 0.84 (1.71–5.90)
FEV_1_ of predicted (%)	94 ± 12 (62–127)	94 ± 12 (71–129)	94 ± 12 (62–129)
FVC (L)	5.28 ± 0.82 (3.09-8.03)	3.65 ± 0.61 (2.15-5.39)	4.34 ± 1.07 (2.15-8.03)
FVC of predicted (%)	99 ± 11 (67–127)	99 ± 12 (72–145)	99 ± 12 (67–145)
FEV_1_/FVC	0.77 ± 0.06 (0.57–0.94)	0.79 ± 0.06 (0.64–0.93)	0.78 ± 0.06 (0.57–0.94)
FEV_1_/FVC of predicted (%)	95 ± 7 (71–113)	95 ± 6 (80–115)	95 ± 7 (71–115)
Bronchodilatation from baseline, ΔFEV_1_ (%)	3.1 ± 3.5 (−4–21)	2.0 ± 2.9 (−5–15)	2.4 ± 3.2 (−5–21)

**Table 2:** Smoking, allergic sensitization, respiratory symptoms and asthma, *n* = 292. Figures indicate numbers of subjects and their percentage in the groups.

	Men (*n* = 123)	Women (*n* = 169)	Total (*n* = 292)
Non-smokers, *n* (%)	47 (38.2)	74 (43.8)	121 (41.4)
Ever-smokers, *n* (%)	76 (61.8)	95 (56.2)	171 (58.6)
1 positive SPT reaction, *n* (%)	55 (50.0)	63 (44.7)	118 (47.0)
≥6 positive SPT reactions, *n* (%)	10 (9.1)	5 (3.5)	15 (6.0)
Allergic rhinoconjunctivitis (ARC), *n* (%)	40 (32.5)	71 (42.0)	111 (38.0)
Family history of asthma, *n* (%)	17 (13.8)	36 (21.3)	53 (18.2)
Physician-diagnosed asthma, *n* (%)	7 (5.7)	6 (3.6)	13 (4.5)
Asthma medication ever, *n* (%)	21 (17.1)	31 (18.3)	52 (17.8)
Asthma medication past 12 months, *n* (%)	9 (7.3)	13 (7.7)	22 (7.5)

Skin-prick tests (SPTs) were performed for men (*n* = 110) and women (*n* = 141) <61 years (*n* = 251).

The representativeness of the present study cohort was compared with that of the original study cohort of FinEsS I postal survey by gender and age, and use of the replies to the postal questionnaire (6). In the present study cohort, physician-diagnosed chronic bronchitis and symptoms related to chronic bronchitis were less frequently reported than in the original study cohort (FinEsS part I). The prevalences of reported respiratory symptoms, symptoms of allergic rhinoconjunctivitis (ARC), physician-diagnosed asthma and smoking were alike.

### Clinical visits

The BHR challenge test was carried out within 14 days after the initial clinical visit. Of those 310 subjects who participated and were assigned according to the protocol for the bronchial provocation test, 18 subjects did not fulfill inclusion criteria for the baseline FEV_1_.

Among 83 subjects (28%), the histamine challenge test was performed during the period April to June during the main Finnish pollen season. The clinic visit included a structured interview, flow-volume spirometry with bronchodilation test and SPTs. The interview was held by a physician, and spirometry and the SPTs were performed by a trained nurse.

The interview comprised 162 questions on respiratory symptoms, family history of asthma and allergy, living conditions, occupation, and smoking habits (25, 26).

SPTs were performed in subjects <61 years old with allergen extracts for two controls (positive control, histamine 10 mg/mL and negative control, glycerin solvent) and 15 allergens (cat, dog, cow, horse, birch, timothy, mugworth, *Alternaria alternata*, *Cladosporium herbarum*, *Dermatophagoides pteronyssinus*, *Dermatophagoides farinae*, *Acarus siro*, *Lephidoglyphus destructor*, cockroach, latex) (27). The decision as to the age limit for SPTs was based on earlier data on decreased skin reactivity in later adult life (28).

Subjects underwent flow-volume spirometry with a Vmax22 Spirometer (SensorMedics, Yorba Linda, CA, USA) according to 1994 American Thoracic Society criteria (29), wearing nose clips. We measured bronchodilatation response after their inhalation of salbutamol aerosol (0.4 mg) via a spacer (Ventoline®; GlaxoSmithKline, Brentford, UK). We recorded the largest FEV_1_ and FVC from at least three acceptable curves.

Inclusion criteria for the BHR tests were a pretest value of FEV_1_ ≥ 60% of predicted or ≥1.5 L, no respiratory infection within 4 weeks prior to the tests, no severe heart diseases (myocardial infarction within 3 months, unstable coronary disease, dysfunction, arrhythmia), and no stroke. Subjects could use their regular medication, except β-agonists (short-acting beta agonist for 12 h, long-acting beta agonist for 48 h), and antihistamines for 5 days.

The bronchial challenge was conducted with histamine by a dosimetric method with controlled tidal breathing by use of the Spira Elektro2 jet nebulizer (Respiration Care Centre Ltd., Hämeenlinna, Finland) (30). Subjects inhaled buffered histamine diphosphate aerosol in fourfold increasing doses (0.025 mg, 0.1 mg, 0.4 mg, 1.6 mg) at 5-min intervals. The end-point of the BHR test was either an at least 15% fall in FEV_1_ or the maximum noncumulative dose of histamine of 1.6 mg. After the provocation, 0.4 mg of salbutamol (Ventoline, GlaxoSmithKline, Brentford, UK) was given via spacer (Volumatic®; GlaxoSmithKline, London, UK). Post-bronchodilatation FEV_1_ was measured 15 min thereafter. PD_15_FEV_1_ value for histamine was calculated by interpolation (31). The dose-response slope (DRS) was calculated as the relationship of the maximum percent decline in FEV_1_ and the relevant dose of histamine by the method of O'Connor *et al*. (32). The challenge test lasted 30 min.

### Definitions

Classification of BHR severity in the present study is based on an earlier clinical validation study of the histamine method (30). For the histamine challenge, the following classification criteria for BHR were: severe, PD_15_FEV_1_ ≤ 0.100 mg; moderate, 0.100 < PD_15_FEV_1_ ≤ 0.400 mg; mild, 0.400 < PD_15_FEV_1_ ≤ 1.600 mg; and no BHR, PD_15_FEV_1_ > 1.600 mg.

BHR: histamine PD_15_FEV_1_ ≤ 1.6 mg, the higher cut-off level (in the regression analysis) for abnormal BHR. Includes subjects with severe, moderate or mild BHR (30).

BHR_ms_: histamine PD_15_FEV_1_ ≤ 0.4 mg, the lower cut-off level (in the regression analysis). PD_15_FEV_1_ ≤ 0.4 mg is regarded and serves as a diagnostic criterion for asthma in Finland (30, 33).

Non-smoker: never a smoker or smoking fewer than four cigarettes per month.

Ex-smoker: those who had quit smoking at least 1 year prior to the study.

Ever smoker: smokers and ex-smokers.

Family history of asthma: subjects who responded ‘yes’ to the postal survey question, ‘Has any of your parents, brothers or sisters had asthma?’(6)

Pollen season/tested in April–June: subjects on whom the BHR test was carried out in April, May or June.

Allergic sensitization/atopy: a positive SPT reaction to at least one single allergen.

Multisensitization: positive SPT reactions to ≥6 single allergens.

ARC: subjects who responded ‘yes’ to the question, ‘Do you have or have you had hay fever or allergic rhinitis or conjunctivitis?’

Physician-diagnosed asthma: subjects who responded ‘yes’ to ‘Have you been diagnosed as having asthma by a physician?’

Asthma medication ever: subjects who responded ‘yes’ to ‘Do you currently use or have you earlier used asthma medicine regularly or as needed?’

Asthma medication past 12 months: subjects who responded ‘yes’ to ‘Have you used any asthma medicines in the last 12 months?’

Severe respiratory infection at age < 5 years: subjects who responded ‘yes’ to ‘Did you have any severe respiratory infection before the age of 5 years, for example, whooping cough or croup?’

Ever-wheezing: subjects who responded ‘yes’ to ‘Have you ever had wheezing or whistling in your chest when breathing?’

Shortness of breath (SOB) in the past 12 months: subjects who responded ‘yes’ to the question, ‘Have you had any attacks of SOB or breathlessness in the last 12 months?’

SOB and wheezing in the past 12 months: subjects who responded ‘yes’ to the question: ‘Have you had any attacks of SOB with wheezing or whistling in the last 12 months?’

SOB and wheezing at night: subjects who responded ‘yes’ to the question, ‘Have you ever been awakened at night or early in the morning by an attack of SOB with wheezing or whistling?’

Childhood wheezer: subjects who responded ‘yes’ to the question, ‘Have you had wheezing or whistling in your chest in early childhood or asthma during childhood?’

### Statistical analyses

Chi-squared and linear-by-linear tests allowed assessment of the effect of demographic categorical variables on the BHR. We determined BHR severity, risk factors and symptoms associated with BHR using two different cut-off levels of PD_15_FEV_1_. Binary logistic regression analysis served to assess the correlation between BHR and answers to the questionnaire. Age, gender, family history of asthma and determinants significant in the univariate analysis were included in the multivariate regression analysis. Correlations and logistic regression of DRS were calculated after ln transformation by the formula ln(DRS + 1), in which 1 served as a constant in order to avoid negative values of DRS. The representativeness of the present cohort was studied by 95% confidence intervals (CIs).

The programs of SPSS (SPSS for Windows version 15.0; SPSS, Inc., Chicago, IL, USA) and StatXact 8-2007 (Cytel Inc., Cambridge, MA, USA) served for statistical analysis.

## Results

**Figure 2:**
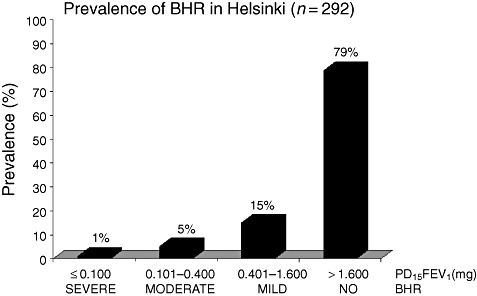
Prevalence of bronchial hyperresponsiveness (BHR) in an adult population in Helsinki assessed with histamine challenge test (Sovijärvi *et al*. (30)). PD_15_FEV_1_, provocative dose inducing a decrease in forced expiratory volume in the first second by 15%.

Of all the subjects, 78.8% had no BHR. The proportion of subjects with BHR was 21.2% (*n* = 62) and with BHR_ms_ 6.2% (*n* = 18) (Fig. 2).

Prevalence of smoking, allergic sensitization and physician-diagnosed asthma are presented in Table 2. Demographic variables, data of asthma, respiratory symptoms and lung function in relation to BHR are shown in Table 3.

**Table 3:** Demographic variables, and data for asthma, respiratory symptoms and lung function of the study cohort (*n* = 292). Univariate risk factors for bronchial hyperresponsiveness (BHR) by odds ratios (ORs) with 95% confidence intervals (CI) in two different cut-off points for histamine provocative dose inducing a decrease in FEV_1_ by 15% (PD_15_FEV_1_) ≤ 1.6 mg and PD_15_FEV_1_ ≤ 0.4 mg

		PD_15_ ≤ 1.6 mg, *n* = 62		PD_15_ ≤ 0.4 mg, *n* = 18	
			
	no. (% of 292)	*N* (% of no.)	OR (95%CI)	*N* (% of no.)	OR (95%CI)
Age					
26 < 41 years	100 (34.2)	20 (20.0)	1	5 (5.0)	1
41 < 53 years	96 (32.9)	18 (18.8)	0.92 (0.45–1.88)	2 (2.1)	0.40 (0.08–2.14)
53–66 years	96 (32.9)	24 (25.0)	1.33 (0.68–2.62)	11 (11.5)	2.46 (0.82–7.36)
Gender					
Men	123 (42.1)	20 (16.3)	1	8 (6.5)	1
Women	169 (57.9)	42 (24.9)	1.70 (0.94–3.08)	10 (5.9)	0.90 (0.35–2.36)
BMI					
>30	48 (16.4)	12 (25.0)	1.29 (0.63–2.67)	3 (6.3)	1.02 (0.28–3.66)
Ventilatory function*					
FEV_1_ ≥ 80% of predicted	259 (88.7)	45 (17.4)	1	8 (3.1)	1
FEV_1_ < 80% of predicted	32 (11.0)	17 (53.1)	5.39 (2.51–11.58)	10 (31.3)	14.26 (5.11–39.82)
FVC ≥ 80% of predicted	279 (95.5)	57 (20.4)	1	18 (6.5)	0
FVC < 80% of predicted	12 (4.1)	5 (41.7)	2.78 (0.85–9.09)	0	0
FEV_1_/FVC < 88% of predicted	37 (12.7)	18 (48.6)	4.52 (2.20–9.31)	9 (24.3)	8.75 (3.21–23.86)
FEV_1_ < 80% of predicted and FEV_1_/FVC < 88% of predicted	11 (3.8)	7 (63.6)	7.16 (2.02–25.32)	7 (63.6)	42.80 (10.89–168.15)
Reversibility in FEV_1_[ΔFEV_1_ (L) + 12% and ≥0.2 L]	4 (1.4)	3 (75.0)	11.64 (1.19–113.98)	1 (25.0)	5.31 (0.52–53.83)
Smoking					
Non-smokers	121 (41.4)	19 (15.7)	1	3 (2.5)	1
Current and ex-smokers	171 (58.6)	43 (25.1)	1.80 (0.99–3.28)	15 (8.8)	3.78 (1.07–13.37)
Family history of asthma	53 (18.2)	14 (26.4)	1.43 (0.72–2.84)	4 (7.5)	1.31 (0.41–4.16)
BHR tested in April–June	83 (28.4)	19 (22.9)	1.15 (0.62–2.11)	9 (10.8)	2.70 (1.03–7.07)
Multisensitization (SPT ≥ 6 allergens) †	15 (6.0)	4 (26.7)	1.42 (0.43–4.67)	3 (20.0)	4.67 (1.16–18.78)
Severe respiratory infection at age <5 years	46 (15.8)	16 (34.8)	2.32 (1.17–4.61)	4 (8.7)	1.58 (0.50–5.03)
Physician-diagnosed asthma ever	13 (4.5)	5 (38.5)	2.43 (0.77–7.23)	3 (23.1)	5.28 (1.31–21.22)
Asthma medication ever	52 (17.8)	17 (32.7)	2.11 (1.08–4.09)	6 (11.5)	2.48 (0.89–6.94)
Asthma medication (past 12 months)	22 (7.5)	11 (50.0)	4.29 (1.76–10.45)	4 (18.2)	4.06 (1.21–13.62)
Symptoms					
Ever wheezing	134 (45.9)	41 (30.6)	2.88 (1.60–5.18)	16 (11.9)	10.58 (2.39–46.90)
Shortness of breath past 12 months	60 (20.5)	23 (38.3)	3.08 (1.65–5.74)	8 (13.3)	3.42 (1.29–9.08)
Shortness of breath and wheezing in the past 12 months	17 (5.8)	13 (76.5)	14.99 (4.69–47.93)	4 (23.5)	5.74 (1.66–19.88)
Shortness of breath and wheezing at night	15 (5.1)	9 (60.0)	6.34 (2.16–18.58)	4 (26.7)	6.83 (1.93–24.19)

* FEV_1_ and FVC values were obtained from 291 subjects. Predicted values according to Viljanen *et al*. (24).

† SPT done for subjects <61 years of age, *n* = 251.

BMI, body mass index; FEV_1_, forced expiratory volume in the first second; FVC, forced vital capacity; SPT, skin-prick test.

### Determinants of BHR

The main determinant for BHR and BHR_ms_ was decreased FEV_1_ (<80% of predicted), odds ratio (OR) 5.39 and 14.26, respectively. The strongest determinant of BHR was FEV_1_ < 80% of predicted combined with airway obstruction (FEV_1_/FVC < 88% of predicted) (OR 7.16, 95% CI 2.02–25.32). Reversibility of the airway defined as FEV_1_ change after bronchodilatation (ΔFEV_1_) + 12% and ≥0.2 L occurred in four subjects, of whom three had BHR (OR 11.64, 95% CI 1.19–113.98). Wheezing, SOB and nocturnal asthma symptoms were significantly associated with BHR and BHR_ms_. (Table 3).

Of the 18 subjects with BHR_ms_, 10 (56%) had baseline FEV_1_ below the normal range, of whom 7 also had decreased FEV_1_/FVC (<88% of predicted) with an OR of 42.80 for BHR_ms_. Ever-smokers were at risk for BHR_ms_ (OR 3.78).

We found no association of age, gender, body mass index or family history of asthma with BHR. Cold air- or exercise-induced symptoms were not significantly associated with BHR of the magnitude tested. Neither rural living vs urban, childhood conditions, number of siblings, day care before school age, history of eczema nor pets at home were significantly associated with BHR.

Physician-diagnosed asthma was reported by 13 subjects (4.5%), of whom three had FEV_1_ < 80% of predicted and nine were ever-smokers. Physician-diagnosed asthma was associated with BHR_ms_ (OR 5.28).

Only five subjects (1.7%) had used inhaled or cortisone per os on the day of testing. Any physician-prescribed asthma medication ever taken was the response of by 52 subjects (18%), of whom 22 (42%) had used asthma medication during the past 12 months. Any use of inhaled corticosteroids was associated with BHR (OR 6.05) and even to a greater extent with BHR_ms_ (OR 12.76).

Of the subjects tested, 17 (5.8%) reported asthma or wheezing in childhood; all had a normal FEV_1_ (≥80% of predicted), seven subjects (41%) had BHR, three had used asthma medication during the past 12 months, and two had both BHR and physician-diagnosed asthma. Severe respiratory infection before age 5 was associated with BHR (OR 2.32).

**Figure 3:**
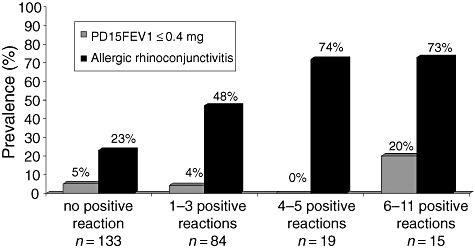
Frequency distribution of moderate or severe bronchial hyperresponsiveness (BHR_ms_) [provocative dose inducing a decrease in forced expiratory volume in the first second by 15% (PD_15_FEV_1_) ≤ 0.4 mg] and allergic rhinoconjunctivitis (ARC) in four categories of allergic sensitization in terms of positive reactions in skin-prick tests. This data is obtained from subjects <61 years (*n* = 251). For BHR_ms_, the Spearman two-tailed association, *P* = 0.018 and one-way ANOVA *P* = 0.058; linear-by-linear test as the test for trend of symptoms of ARC, *P* < 0.001.

Neither atopy nor positive reaction to any single allergen tested correlated with BHR. Atopy combined with obstruction (FEV_1_/FVC < 88% of predicted), however, led to increased risk for BHR (OR 6.32). Multisensitization was associated with BHR_ms_ (OR 4.67) (Fig. 3), whereas respiratory symptoms of food allergy – SOB or wheezing – associated only with BHR (OR 6.67).

Subjects who took the bronchial provocation test during the pollen season (April–June) showed no difference in reporting rhinoconjunctivitis from those tested outside the pollen season. Those tested during the season, however, were at increased risk for BHR_ms_ (OR 2.70). Of the nonatopic subjects, 30 (23%) reported symptoms of ARC, 28 (21%) had BHR, of whom nine (32%) had BHR_ms_.

### Multivariate relationships

The independent determinants for BHR_ms_ were FEV_1_ < 80% of predicted (OR 27.18), FEV_1_/FVC < 88% of predicted (OR 6.16) and use of asthma medication ever (OR 6.72). Whereas risk factors for BHR were decreased FEV_1_ (OR 4.09), FEV_1_/FVC < 88% of predicted (OR 4.33), history of severe respiratory infection before age 5 (OR 2.65), and reported SOB and wheezing in the past 12 months (OR 13.00) (Table 4).

**Table 4:** Risk in odds ratios (ORs) with 95% confidence intervals (CI) for histamine provocative dose inducing a decrease in FEV_1_ by 15% (PD_15_FEV_1_) ≤ 1.6 and PD_15_FEV_1_ ≤ 0.4 mg, according to multivariate analysis, all subjects (*n* = 292) included

	PD_15_FEV_1_ ≤ 1.6 mg	PD_15_FEV_1_ ≤ 0.4 mg
	OR (95%CI)	OR (95%CI)
Age		
26 < 41 years	1	1
41 < 53 years	0.91 (0.38–2.20)	0.67 (0.08–5.40)
53–66 years	1.13 (0.45–2.84)	2.79 (0.43–17.94)
Gender		
Men	1	1
Women	1.87 (0.85–4.10)	1.86 (0.35–9.85)
Family history of asthma	0.96 (0.37–2.48)	0.22 (0.02–2.04)
Ventilatory function		
FEV_1_ < 80% of predictive	4.09 (1.45–11.52)	27.18 (4.91–150.57)
FEV_1_/FVC < 88% of predictive	4.33 (1.69–11.06)	6.16 (1.18–32.21)
Multisensitization (SPT ≥ 6 pos)	0.81 (0.16–4.07)	7.33 (0.69–77.63)
BHR tested in April–June	0.63 (0.26–1.48)	1.00 (0.18–5.58)
Childhood		
Severe respiratory infection <5 years	2.65 (1.05–6.70)	2.00 (0.25–16.25)
SOB and wheezing in the past 12 months	13.00 (2.64–63.91)	2.29 (0.25–20.91)
Asthma medication ever	1.83 (0.72–4.67)	6.72 (1.12–40.53)
Smoking		
Non-smokers	1	1
Current and ex-smokers	1.07 (0.48–2.37)	1.07 (0.14–8.11)

FEV_1_, forced expiratory volume in the first second; FVC, forced vital capacity; SOB, shortness of breath; SPT, skin-prick test.

When multisensitization and obstruction (FEV_1_/FVC < 88% of predicted) were analyzed together, they both remained independent risk factors for BHR_ms_ (OR 4.68, 95% CI 1.01–21.65 and OR 8.29, 95% CI 2.47–27.81, respectively). These ORs present the same level of increased risk as calculated in univariate analysis.

### DRS

LnDRS correlated significantly with BHR and BHR_ms_ (Spearman correlation, coefficients 0.664 and 0.415, *P* < 0.001 for both), and lnDRS associated significantly with use of asthma medication in the preceding 12 months (*P* = 0.028), age (*P* = 0.027) and FEV_1_ below predicted (*P* = 0.042).

## Discussion

BHR occurred in 21%, and 6% presented BHR_ms_. The latter is in line with the reported prevalence of physician-diagnosed asthma (7%) in Helsinki (6). We found no discrepancy with results of the European Community Respiratory Health Survey in which the prevalence of BHR ranged from 3% to 28% among 16 countries, with a median prevalence of 13% (34).

We assessed BHR by two cut-off levels of PD_15_FEV_1_ indicating different severity levels of BHR, thus showing variations and differences in importance of the determinants assessed. For most of the determinants, ORs were higher, parallel with BHR severity. Our results, however, revealed that some risk factors for BHR and asthma, like severe respiratory infection in childhood, were associated with the higher PD_15_FEV_1_ cut-off level only.

The strongest determinant of BHR was decreased FEV_1_ (<80% of predicted) when combined with airway obstruction (defined as FEV1/FVC < 88% of predicted). But, studies on the association of BHR with allergic sensitization have yielded a variety of results (35–37). In the present study, atopy only if combined with obstruction in the spirometry led to increased risk for BHR.

Multisensitization independently led to increased risk also for BHR_ms_. The results on BHR_ms_ agree with findings in experimental studies where the site of obstruction was assessed with synchrotron lung function imaging after methacholine and ovalbumin in sensitized rabbits (38). Provocation with the allergen caused peripheral constriction in the airway different from the more proximal airway constriction caused by intravenous metacholine. However, inhaled methacholine caused a more peripheral bronchoconstriction and a markedly heterogenous ventilation. This finding can, in part, explain our findings with enhanced bronchial responsiveness to inhaled histamine in subjects with decreased FEV_1_ and FEV_1_/FVC, and with multisensitization.

We found that one out of five nonatopic subjects reported symptoms typical for ARC and also had BHR. In this study, the spring pollen season (April–June), regardless of atopic status, elevates risk for BHR_ms_, which indicates that some undefined environmental factors are linked with BHR. The airway smooth muscle cells are strongly involved also in noneosinophilic airway inflammation (39). The impact of time and season of testing may explain intrasubject variation in degree of BHR, a fact to consider in clinical use and in treatment strategies.

The present results confirm that smoking is a risk factor for BHR and BHR_ms_(40). However, in the multivariate analysis, its statistical significance vanished. Further detailed research is needed to assess relation of smoking habits and BHR.

In this study, female gender and BHR showed no association in contrast with some other's findings (8, 41, 42). The explanation may, at least in part, depend on differences in cohorts’ lung-function values or the use of that data. We used in the analyses spirometric values of predicted, which normalizes the values by gender, age and height. Thus, in this study, we could not assess the gender effect.

The present study has certain limitations mostly because of relatively small number of subjects, slightly below 300. We assessed the representativeness of the present study cohort. The replies concerning the main risk factors for BHR did not reveal discrepancy in the representativeness of the smaller cohort of the present study in comparison with the original study cohort of randomly selected 6062 Helsinki inhabitants.

Because of many differences between study cohorts, such as differences in inclusion criteria and variety of absolute lung volumes, the prevalence of BHR remains arbitrary. Here, 18 subjects (6%) were excluded from BHR measurements because of a low FEV_1_ (<60% of predicted), which may underestimate the real magnitude and prevalence of BHR. This fact has received little attention in the interpretation or discussion of any of the BHR general population studies. BHR prevalence studies, as the present investigation, often miss some severe asthma patients because of exclusion criteria by FEV_1_, thus diminishing the prevalence of physician-diagnosed asthma in the cohort. Final results will then lack these patients’ data on reported associations, risk factors and determinants of BHR.

The use of asthma medication reduces or abolishes BHR (43). The real influence of medication on the results is difficult to assess in an epidemiologic study setting. We found that asthma diagnosis and medication was associated with BHR, but most of the subjects with BHR_ms_ were those without physician-diagnosed asthma or those without asthma treatment during the past 12 months. We believe that because of the asthma treatment strategies adopted in Finland (33), subjects using asthma medication and with physician-diagnosed asthma were not those who had severe BHR in the present study. In the present cohort, 4.5% of the subjects reported physician-diagnosed asthma, which is in line with the percentage of those receiving special reimbursement of asthma drugs (4.1%) based on chronic asthma in Finland in 2004 (33).

The clinical interview of the present study yielded valuable data from these subjects’ childhoods but offered no simple answer to the question as to use of inhaled corticosteroid treatment for wheezing (44, 45). Our results indicate that in adult subjects with symptoms of asthma in childhood, risk for BHR is higher. Determination of BHR in these subjects with severe respiratory symptoms in childhood may be clinically useful when they start to complain about respiratory symptoms in adulthood.

The use of DRS has been suggested for epidemiologic studies in which a majority of those studied show no BHR (46). Here, the results of DRS analysis provided no additional value to results obtained by PD_15_FEV_1_.

In this adult general population of Helsinki, 79% of the subjects did not present with BHR (PD_15_FEV_1_ > 1.6 mg). This is in line with the high negative value of the histamine PD_15_FEV1 > 1.6 mg for asthma with the presently used dosimetric histamine method (30, 47). The prevalence of respiratory symptoms among those without BHR seems to be common; the majority of those who reported symptoms were subjects without BHR.

## Conclusions

Of the adult population of Helsinki, 21% showed BHR to inhaled histamine. Decreased FEV_1_ and airway obstruction were the main determinants for BHR. Ever-smoking, multisensitization and the examinations taking place during the pollen season were significantly associated with BHR_ms_, whereas a severe respiratory infection in childhood was associated with generally increased BHR only. Use of asthma medication associated with increased BHR and the prevalence of BHR_ms_ were similar to the reported prevalence of physician-diagnosed asthma in Finland. Quantitative assessment of BHR by different cut-off levels provides a tool for characterization of phenotypes of airway disorders in epidemiologic and clinical studies.
